# Climate variability and migration in the Philippines

**DOI:** 10.1007/s11111-016-0263-x

**Published:** 2016-10-08

**Authors:** Pratikshya Bohra-Mishra, Michael Oppenheimer, Ruohong Cai, Shuaizhang Feng, Rachel Licker

**Affiliations:** 1 Woodrow Wilson School of Public and International Affairs, Program in Science, Technology and Environmental Policy, Princeton University, Princeton, NJ 08544 USA; 2 Woodrow Wilson School of Public and International Affairs, Program in Science, Technology and Environmental Policy, and Department of Geosciences, Princeton University, Princeton, NJ 08544 USA; 3 Global Climate Program, Environmental Defense Fund, New York, NY 10010 USA; 4 Institute for Economic and Social Research, Jinan University, Guangzhou, 510632 China

**Keywords:** Climate change, Migration, Environmental migrants, Temperature, Typhoons, Agricultural productivity

## Abstract

This study investigates the effects of climatic variations and extremes captured by variability in temperature, precipitation, and incidents of typhoons on aggregate inter-provincial migration within the Philippines using panel data. Our results indicate that a rise in temperature and to some extent increased typhoon activity increase outmigration, while precipitation does not have a consistent, significant effect. We also find that temperature and typhoons have significant negative effects on rice yields, a proxy for agricultural productivity, and generate more outmigration from provinces that are more agriculturally dependent and have a larger share of rural population. Finally, migration decisions of males, younger individuals, and those with higher levels of education are more sensitive to rising temperature and typhoons. We conclude that temperature increase and to some extent typhoon activities promote migration, potentially through their negative effect on crop yields. The migration responses of males, more educated, and younger individuals are more sensitive to these climatic impacts.

## Introduction

In the context of interactions between humans and the natural environment, migration has been identified as a key demographic response to environmental changes. Although qualitative studies have traditionally dominated the literature on this topic, the past decade has witnessed a surge in the volume of quantitative studies that explore the influence of rainfall, temperature, and natural disasters on both internal and international migration.^1^ In general, studies have found that temperature variations and episodic disasters have a significant positive effect on short-distance internal migration. Evidence on the effect of rainfall on internal migration is mixed. Few studies have simultaneously examined the effects of both disasters and climatic variations on migration, and typically, studies use only variation in precipitation as a measure of climatic variation. Since precipitation and temperature are correlated, both should be included in a model to derive an unbiased effect of either one on migration (Auffhammer et al. 2013).

The primary focus of this paper therefore is to explore the effects of both climatic variations and extremes on aggregate inter-provincial migration flows in the Philippines using data on annual variations in the incidents of typhoons, along with variability in precipitation and temperature. By doing so, we attempt to improve upon the existing studies and more accurately predict the role of climatic factors on internal migration. This paper is also motivated by previous findings that suggest agriculture is a potential channel for climate-induced migration (Feng et al. 2010; Feng and Oppenheimer 2012; Cai et al. 2016; Mueller et al. 2014). The study adds to the literature by exploring the underlying mechanism for climate-induced migration through an exploration of the link between crop yields and climatic variations. Finally, we explore the climate–migration sensitivity to demographic and socioeconomic characteristics, an aspect examined in only a few previous studies. We attempt to fill this gap by predicting the effects of climatic factors on migration outcome among different demographic groups broken down by gender, age, and education.

We base our study in the Philippines for a number of reasons. As an archipelago of 7107 islands, the population of the Philippines is highly exposed to climatic variations, extreme climatic events, and climate-related hazards such as frequent typhoons, rising sea levels, and the resulting risk in flooding. Additionally, a large portion (33 % in 2011)^2^ of the population is employed in the agricultural sector—an economic sector that is directly reliant on environmental and climatic conditions. Furthermore, an estimated 60 %^3^ of the population resides near coastlines. All these factors make the population extremely vulnerable to the impacts of climate change.

In addition, the Philippines lies in a region that experiences some of the highest typhoon activity in the world. An average of five typhoons make landfall in the country every year, resulting in flooding, landslides, property damage, and loss of human life (Huigen and Jens 2006; and Kubota and Chan 2009). Therefore, the Philippines provides a unique opportunity to study the demographic response to frequent natural disasters—captured here by temporal variation in typhoon activity—as opposed to rarely occurring disastrous events such as volcanoes or earthquakes.

The Philippines is a country with a high level of internal migration (Quisumbing and McNiven 2005). According to Quisumbing and McNiven (2005), and Gultiano and Xenos (2004), internal migration in the Philippines is largely rural to urban and rural to rural. Recent migration flows are revealed to be mostly interprovincial, typically in the direction of metropolitan Manila and surrounding areas (Quisumbing and McNiven 2005). Furthermore, internal migration rate is found to be the highest among women and youth, especially from 1960s onwards (Quisumbing and McNiven 2005, and Gultiano and Xenos 2004). In terms of education, based on research conducted in the mid-1980s as well as 2003–2004, more educated individuals migrated to poblaciones^4^ and cities for employment or education, while less educated men were likely to migrate to nearby urban areas to engage in manual labor such as construction work (Deshingkar and Natali 2008).

Given the high level of exposure and vulnerability to climate hazards, the evidence for populations to respond to climatic variations by migrating in other parts of the world, as well as the significance of internal migration in the Philippines, we examine the sensitivity of recent internal migration flows in the country to climate variations. We find that rising temperature and to some extent typhoon activity promote internal migration, potentially through their negative effects on crop yields based on our findings on the effects of climate variability on rice yield. Precipitation, however, has no significant effect on migration. We further find that, in general, males, more educated, and younger individuals are more sensitive to such climate variability.

## Literature review and theoretical framework

A detailed review of the existing literature on environmental migration reveals that, by and large, temperature variations and disastrous events induce short-distance internal migration, but the findings on the impact of rainfall on internal migration are mixed. Among a number of studies investigating the link between rainfall and internal migration, reduced rainfall is linked to increased migration to urban areas in sub-Saharan Africa (Barrios et al. 2006); an increase in internal migration within Tanzania (Afifi et al. 2014); and an increase in short-distance moves in the context of Mali and Burkina Faso (Findley 1994 and Henry et al. 2004). Periods of extended droughts have also been shown to promote internal migration due to a decline in soil quality for farming (McLeman and Ploeger 2012).

A number of other studies find no significant effect of precipitation on internal migration. For example, Mueller et al. (2014) find no robust effect of rainfall on within or across village migration of men or women in Pakistan. Gray and Bilsborrow (2013) find that rainfall was the least important predictor of internal moves in rural Ecuador. In Ghana, Abu et al. (2014) show that rainfall did not have a major impact on the migration intentions of those in agricultural communities although the majority perceived rainfall as their most pressing stressor given their dependence on agriculture.

With regard to the effect of temperature on migration, only a small number of studies at the microlevel^5^ (Mueller et al. 2014; Bohra-Mishra et al. 2014) and a handful of macrolevel^6^ studies (Collier and Hoeffler 2011; Beine and Parsons 2012; Feng et al. 2010; Feng and Oppenheimer 2012; Feng et al. 2013; Cai et al. 2016) investigate the relationship. Furthermore, even fewer studies investigate the link between temperature and internal migration. Among the studies that focus on internal migration, Feng et al. (2013) show that in the US Corn Belt, a further rise in baseline temperature increases internal, across-county migration through its effect on crop yields; Bohra-Mishra et al. (2014) find that in Indonesia, which has a relatively high baseline temperature, a further increase in temperature increases inter-provincial migration of households; and Mueller et al. (2014) show that higher temperature in the Rabi season consistently increases migration of men to other villages. Similarly, Poston et al. (2009) find that within the USA, the climate (captured by measures of temperature, humidity, and wind) of sending as well as receiving states had a significant positive impact on aggregate interstate migration, such that states with more favorable climate received more in-migrants and vice versa.

Disasters are also likely to influence internal migration. In general, following natural disasters, people appear to use their social network and tend to move to their closest kin, yielding movements that are local or short distance because they look for the nearest safe location. Utilizing global panel data of bilateral migration flows, Beine and Parsons (2012) find a positive role of disasters on internal migration to urban areas. Consistent with this finding, existing microlevel studies generally conclude that natural disasters result in short-distance internal migration (Lu et al. 2012; Haque 1986; Rofi et al. 2006; Hassani-Mahmooei and Brett 2012; Martin et al. 2014; Dun 2011; Afifi et al. 2014; Salauddin and Ashikuzzaman 2012; Gray and Mueller 2012). Based on these existing findings, we hypothesize that disasters are likely to influence internal migration, especially given the evidence that in the aftermath of disasters people utilize their social network and migrate to the nearest safe location. Similarly, based on existing evidence, in places with relatively high baseline temperature, a further rise in temperature is likely to motivate outmigration. We therefore hypothesize that in tropical places like the Philippines, which already experience relatively high temperature, a further increase in temperature promotes internal migration. On the other hand, the link between precipitation and migration is unclear in earlier work and we have no specific expectation in the Philippines case.

If climatic variations indeed influence internal migration, what could be the underlying mechanism that could explain such a link? Climate may influence migration through economic channels. At a macrolevel, Dell et al. (2008) find that higher temperatures in a given year reduce the growth rate of GDP per capita in poor countries. Feng et al. 2010, Feng and Oppenheimer 2012, and Cai et al. 2016 find that climate variability increases outmigration through its negative impact on agricultural productivity. Rising temperature, in particular, could influence outmigration through a negative effect on income in agricultural sector (Mueller et al. 2014; Bohra-Mishra et al. 2014). Studies have found that in regions with a high baseline temperature, rising temperatures tend to negatively affect crop yields (Lobell and Asner 2003; Peng et al. 2004; Schlenker and Roberts 2009; Schlenker and Lobell 2010) as well as agricultural exports in developing countries (Jones and Olken 2010).

Studies similarly show that the productivity of rice, the most important crop in the Philippines, is influenced by increasing temperatures and changes in precipitation patterns due to climate variability. El Niño events in the Philippines, which result in unusually warm temperatures, have been linked to declines in rainfed rice yields (Lansigan 2005; Lansigan et al. 2000; Buan et al. 1996). Peng et al. (2004) demonstrate that rice yield in the Philippines declined by 10 % for each 1 °C increase in growing-season minimum temperature in the dry season, while maximum temperature had no significant impact on yields. Similar findings on the important role of minimum temperature were revealed in Welch et al. (2010) using data from 227 intensively managed irrigated rice farms in six important rice-producing countries in Asia. In terms of the effect of mean temperature, the findings from Peng et al. (2004) imply that rice yield in the Philippines declined by 15 % for each 1 °C increase in growing-season mean temperature.

According to Porter et al. (2014), regardless of the greenhouse gas emissions scenario and implementation of adaptation strategies, tropical countries like the Philippines are very likely (90–100 % probability) to experience negative crop yield impacts by the end of the twenty-first century as a result of anthropogenic climate change. If a large proportion of a country’s population relies upon agriculture as a primary source of livelihood, a potential negative effect of climatic variations on crop yields may motivate migration of people in response to both previous and current impacts on crop yields as well as a strategy to insure against risks from future climate events or trends. According to the theory of new economics of labor migration, households in rural areas of developing countries use migration as a strategy to insure themselves against risks to income (Massey et al. 1998), which in the case of farming households could be the risk from unforeseen climatic events that may result in crop failures. Given the significance of agriculture in the Philippines, we therefore hypothesize that a potential negative effect of climatic variations and extremes on crop yields in turn may influence both past and future migration flows through this agricultural channel. Furthermore, the migratory response to changing climate is likely to vary across demographic and socioeconomic subgroups.

Studies that explore the climate–migration sensitivity by demographic and socioeconomic characteristics are, however, limited. Studies that compare the sensitivity of females’ migratory response to climate variability compared to males’ find inconclusive results that seem to vary by study area. While Feng et al. (2013) find similar sensitivities for men and women in the Midwest Region of the USA, in the context of developing countries however, Mueller et al. (2014) and Gray and Mueller (2012) find that high temperature and drought, respectively, increased male migration as opposed to female migration. Given the existing findings, for a developing country like the Philippines with patriarchal norms, we hypothesize that climatic variations induce male migration more than female migration, primarily because males are the primary bread earners, and for this reason, if there is indeed an economic or agricultural channel through which climate affects migration, males’ migratory response is likely to be affected more than females’.

As for the breakdown by age, typically, migration studies have established a nonlinear effect of age. Among adults, migration increases at earlier years because the returns to age and experience are expected to rise in the earlier years but then declines as they accumulate, which is reflected by reduced migration at older age (Bohra and Massey 2009; Piotrowski 2013). Similarly, in the context of climate variables, the evidence on sensitivity of migration of different age groups suggests a higher migration among younger than older age groups. Feng et al. (2013) find that individuals evince higher sensitivity to climate at younger ages and Abu et al. (2014) find that in the agricultural communities of Ghana where sources of livelihood are affected by environmental stress, older household heads report significantly lower migration intentions compared to younger household heads.

Finally, migration studies have established that financial, social, and human capital (level of education) are important determinants of migration, with migrants having more financial resources, as well as social and human capital relative to the general population to support their moves (Massey and Espinosa 1997; Bohra and Massey 2009). In the context of climate variability, those people who would qualify as non-migrants under normal circumstances are also the most vulnerable to the effects of climate variability. They are therefore impacted the most by variations in climate, which in turn can lower their already limited financial capital, further reducing their ability to use migration as an adaptation strategy.

Consistent with the above theory, previous studies have shown that environmental changes, particularly natural disasters in some situations, such as the case of the poor and vulnerable, may actually constrain movement (Halliday 2008; Yang 2008; Naik 2009; Foresight 2011; Tse 2012, Juelich 2011). In their comparison of migration patterns of individuals from damaged areas versus areas not directly affected by the 2004 tsunami in Indonesia, Gray et al. (2014) find that from both types of areas, educated individuals were more likely to migrate.

We therefore hypothesize that individuals belonging to higher socioeconomic status are more responsive to climatic variations than those from lower socioeconomic status. Additionally, consistent with self-selected voluntary migration of individuals with higher level of human capital, more educated individuals might be more likely to migrate in response to environmental changes since the potential gains from migration for them will be higher. Our paper contributes to the limited existing literature that simultaneously examines the effects of natural disasters and climatic variations on migration and advances the literature by adding to the findings that rising temperature and increased typhoon activity promote migration, potentially through their negative effects on crop yields. The paper further adds to the limited findings on climate–migration sensitivity by demographic groups, leading us to conclude that males, more educated, and younger individuals exhibit a more sensitive migration response to such climatic impacts.

## Data and methodology

Data for migration comes from the 2000 Philippines Census of Population made available by the Integrated Public Use Microdata Series, International (IPUMS).^7^ The dataset provides individual responses to a query on province of residence 5 and 10 years back (as opposed to current province of residence). Using this information, we compared the previous province of residence with the current province locations to establish whether or not an individual migrated during 5-year intervals, 1990–1995 and 1995–2000. We then used the individual migration data to generate aggregated province-to-province migration flows in order to calculate aggregate provincial migration rates. Following the usual practice in the migration literature, we only looked at adults aged 15–65 years (the most active age group) at the beginning of each migration period for our analysis and ignored children and older people. We thus calculated the percentage of people aged 15–65 years old living in each province at the beginning of each 5-year time period who migrated within that time period.

We supplemented the province-level migration data with province-level rice yield data for the Philippines, obtained from the Philippines Bureau of Agricultural Statistics (CountrySTAT)^8^ as well as Ricestat.^9^ Using a standard approach, we divided the data on rice production (in metric ton) by the total production area (in hectare) to generate rice yield data (metric ton per hectare) for each province, which we later converted to kilogram per hectare. The climate variables were derived from data provided by the University of Delaware (Willmott et al. 2010). We used monthly, gridded reconstructions of temperature and precipitation available at a 0.5° × 0.5° resolution. To generate province-level values, we aggregated the respective grid cells that fell within the bounds of each province. For each grid cell, we identified its area under cultivation using a global, gridded dataset of year 2000 area harvested values from Monfreda et al. (2008). As we were interested in isolating the climate over croplands (versus over other land cover types), we weighted the contribution of each grid cell’s climate characteristics to a provincial average climate based on the portion of the grid cell that was harvested. In other words, we produce average, monthly, province-level climate characteristics that represent conditions over areas important for crop production. Finally, we used these monthly mean values to generate annual average, maximum, and minimum of temperature (°C) and precipitation (meters per year from millimeters per year for ease of interpretation) values for each 5-year time period.

Data for measures of typhoon activity were derived from the DesInventar database,^10^ which provides statistics on effects of natural disasters using preexisting official data, academic records, newspaper sources, and institutional reports. The major recurring climate-related disaster that affects the Philippines is typhoons, and therefore, we include the effect of typhoons on migration.

We extracted the above-mentioned proxy measures of typhoons, which were only available at the regional level and assigned them to the provinces (normalized by the provincial population) based on the region each province fell under (see discussion of allocation among provinces below). We then used a logarithmic transformation of the normalized proxy measure of typhoons in order to correct for its positively skewed distribution and therefore correct for any bias in the results driven by extreme values. Although finer-scale provincial-level data for typhoons would have been ideal, these measures allow us to capture both the frequency and the intensity of typhoon activity influencing each province. Following the convention of using deaths as a measure of negative events such as disasters and conflict (see Mueller et al. 2014, Bohra-Mishra and Massey 2011), we choose deaths as a proxy measure of typhoon and use it across all specifications. Using alternate measures of typhoons such as injuries and houses damaged allowed us to compare and confirm results, which we in turn used as a robustness check (results using injuries and houses damaged available upon request). Table 1 presents descriptive statistics of the data described in this section.

**Table 1:** Descriptive statistics

	1990–1995				1995–2000			
	Min	Max	Mean	SD	Min	Max	Mean	SD
Migration								
Domestic outmigration rate (%)	0.01	0.05	0.02	0.01	0.01	0.05	0.02	0.01
Rice yield								
Average total rice yield (kg/Ha)	1110.84	4027.10	2683.56	701.53	1289.94	4041.10	2744.95	642.21
Average wet season rice yield, July–December (kg/Ha)	1267.67	4162.23	2701.55	700.03	1489.77	4334.66	2731.24	624.39
Average dry season rice yield, January–June (kg/Ha)	862.59	4762.00	2706.55	778.88	1089.47	4339.62	2801.71	731.96
Climate variables								
Average summer temperature, April–September (°C)	21.32	28.33	26.27	1.38	20.85	28.58	26.36	1.48
Average of maximum monthly precipitation in the wet season, July–December (m/yr)	0.19	0.78	0.44	0.14	0.24	0.82	0.48	0.13
Average of minimum monthly precipitation in the dry season, January–June (m/yr)	0.00	0.16	0.05	0.04	0.00	0.17	0.06	0.04
Deaths from typhoons (normalized by population of province)	0.00	69.64	4.71	11.81	0.00	3.49	0.52	0.73

Out of a total of 81 provinces in the Philippines, several provinces such as Basilan, Batanes, Camiguin, Romblon, Southern Leyte, and Sulu were missing data on climate, while provinces such as Shariff Kabunsuan, Zamboanga Sibugay, Metropolitan Manila, Tawi–Tawi, and Compostela Valley were missing data on either migration or rice yield. Upon merging all the different datasets, we therefore end up with 69 provinces, which have data on climate, migration, and yield. In our final analysis, we therefore use data from these 69 provinces.

To detect the impact of climatic variations and extremes, including the effect of typhoons on inter-provincial migration, we exploited our panel data structure. This allowed us to use random variations in weather patterns and incidents of typhoons over time that can be treated as exogenous shocks that individuals are exposed to while at the same time controlling for time-invariant characteristics of the provinces, which enables us to establish a plausibly causal link between climatic variations and migration. Our empirical model predicts the effects of variations in average temperature, precipitation, and typhoon activity measured over the period that coincides with the period for which variations in aggregate inter-provincial migration rates are observed, as captured in the equation below:

$$M_{\text{pt}} = \alpha T_{\text{pt}} + \beta_{1} R{\text{Max}}_{\text{pt}} + \beta_{2} R{\text{Min}}_{\text{pt}} + {\varvec{\uplambda}}C_{\text{pt}} + P_{p} + t_{t} + \varepsilon_{\text{pt}}$$

where *M*_pt_ is the percent of the population in the original province *p* at the beginning of time period *t* that migrated to another province during time *t*, where *t* is defined as periods 1990–1995 and 1995–2000. *T*_pt_, *R*Max_*pt*_, *R*Min_pt_, and *C*_pt_ represent summer temperature, maximum monthly wet season precipitation, minimum monthly dry season precipitation, and log of normalized death rates from typhoons in province *p* at time *t*. Coefficient *α* captures the effect of temperature, whereas *β*_*1*_ and *β*_*2*_ capture the effects of rainfall. Our choice of climatic variables is driven by both theory and evidence in the existing literature. For temperature, we used five-year averages of annual “boreal summer” (April to September) temperature since recent findings suggest that when temperature is already hot, a further increase can motivate people to move out (Mueller et al. 2014; Bohra-Mishra et al. 2014). For rainfall, we used two measures to capture extremes in precipitation as we wanted to understand the effects of precipitation on migration at both extremes. Thus, the measures were created based on the two distinct seasons in the Philippines: the wet season (July to December) and the dry season (January to June). We generated 5-year averages of maximum monthly precipitation for the wet season, which captures the high end of precipitation throughout the year and 5-year averages of minimum monthly precipitation for the dry season, which should capture the low end of precipitation throughout the year. *λ* represents the effect of typhoons using number of deaths (normalized by provincial population) during period *t* in province *p*. Consistent with the outcome variable, the aggregate migration rate, which is measured at the province level, all climate variables were also measured or assigned at the province level.

Provincial migration rates may be influenced by province-level characteristics. For example, according to the neoclassical economics theory, people respond to geographic wage differentials and therefore provinces that are more developed and can offer higher wages are likely to attract migrants and vice versa. We therefore controlled for province fixed effects with $P_{p}$, which captures time-invariant, province-specific factors that are likely to be correlated with climate variables and may also influence migration. These factors could include province-specific characteristics such as general level of economic development, location such as whether the province is an island province versus landlocked, and history of migration. Finally, we also controlled for time effects by including year fixed effects, represented by *t*. These year fixed effects account for factors that are common across all provinces that may affect migration trends over time, including policy changes, economic cycles, and technological advancements. This also controls for common climatic shocks, including the El Niño–Southern Oscillation (ENSO).

Our time effects variable *t*_*t*_ controls for average effects that are common across all the provinces; however, it is not able to account for any spatial heterogeneity in spatial correlation. Therefore, we report the results using a standard error estimator that corrects for both cross-sectional spatial dependence or spatial autocorrelation across provinces (since neighboring provinces may share spatially correlated disturbances) and temporal autocorrelation within provinces using the method cited in (Hsiang 2010). $\varepsilon_{\text{pt}}$ is the error term.

There are a couple of limitations of our approach that should be noted. For example, the province fixed effects will not control for characteristics of provinces that change over time. Since our analysis uses only two panels covering only two time periods, 1990 to 1995 and 1995 to 2000, we assume that the general characteristics of the provinces will not change much across the two panels. Nevertheless, as with any panel dataset, if there are time-variant province-specific factors that are correlated with environmental variables and both influence migration outcome, this might lead to biased estimates of climatic variables in our results.

Another limitation of our analysis is that our data only include 69 out of 81 provinces and therefore might be subject to the limitations of a small sample size. A third limitation is that the proxy measures of typhoons were not available at a finer, provincial scale but were only available at the regional level (as noted above). We therefore used the following method to derive provincial-level data: if a region experiences 100 deaths from an incidence of typhoon, each province that falls under that region is assigned 100 deaths. We then normalize the number of deaths by the total population of each province at the beginning of each migration period (1990 and 1995, respectively).

A fourth limitation concerns our analysis on climate–migration sensitivity by demographic subgroups. Due to limitations in terms of how the individual-level data are being used in our analyses to build aggregated migration rates for each subgroup, we cannot run interaction effects to show whether the differences across demographic subgroups are statistically significant. Nevertheless, our main purpose behind the analysis on climate–migration sensitivity by demographic subgroups was to demonstrate whether there are differences in the magnitude of the effects of climate across subgroups if there is a statistically significant effect of climate on migration for the particular subgroup and we are able to achieve that through our analysis. Finally, as common with studies using census data, our migration outcome is likely to entirely miss out short-term interim movements of individuals since the available data only capture the initial and final province of residence over a period of 5 and 10 years.

## Results and discussion

Table 2 summarizes results from our regression that predicts the effects of variations in average summer temperature, average maximum and minimum precipitation, and typhoon activity on inter-provincial migration rates. We find that temperature has a significant positive effect on outmigration. Each 1 °C increase in the average summer temperature increases outmigration rate by 0.6 % points. Similarly, increased typhoon activity induces outmigration such that each percentage increase in normalized death rates from typhoons increases migration by 0.15 % points. In order to be able to compare the effects of different climatic variables and better understand their relative impact as well as their ability to explain overall migration, we also include standardized coefficients for all the predictors. From the standardized coefficients, it becomes obvious that temperature has a much stronger impact on migration compared to typhoons: a one standard deviation increase in temperature corresponds to a 1.16 % increase in outmigration, while a one standard deviation increase in normalized death rates from typhoons corresponds to a 0.17 % increase in outmigration. The extremes of precipitation on the other hand do not have significant effects on migration. In general, our results suggest that too much precipitation in the wet season as well as too little precipitation in the dry season induce some migration, but the coefficients are not significant.

**Table 2:** Climate effects on outmigration

Independent variables	All provinces		Provinces with low share of rural population		Provinces with high share of rural population	
	Actual coefficients	Standardized coefficients	Actual coefficients	Standardized coefficients	Actual coefficients	Standardized coefficients
Average summer temperature, April–September (°C)	0.0060***	1.1594***	−0.0022	−0.4217	0.0104***	1.9989***
	(0.002)	(0.383)	(0.002)	(0.426)	(0.002)	(0.453)
Average of maximum monthly precipitation in the wet season, July–December (m/yr)	0.0027	0.0494	0.0161	0.2924	−0.0072	−0.1301
	(0.003)	(0.060)	(0.010)	(0.187)	(0.006)	(0.109)
Average of minimum monthly precipitation in the dry season, January–June (m/yr)	−0.0283	−0.1663	−0.0296	−0.1739	−0.0031	−0.0179
	(0.024)	(0.139)	(0.041)	(0.244)	(0.035)	(0.205)
Deaths from typhoons (normalized by population of province)	0.0015***	0.1671***	0.0011*	0.1259*	0.0022**	0.2458**
	(0.000)	(0.054)	(0.001)	(0.065)	(0.001)	(0.098)
No. of observations	138	138	66	66	64	64
Adjusted *R*-squared	0.978	0.791	0.986	0.868	0.966	0.667

Migration and climate/disaster variables are both measured as 5-year averages between the time periods of 1990 to 1995 and 1995 to 2000. Logarithmic transformation of measure of typhoons used. Results control for province and time (5-year) fixed effects. Standard errors (in parentheses) corrected for spatial autocorrelation and within province clustering

*** *p* < 0.01; ** *p* < 0.05; * *p* < 0.1

The results are consistent with the existing findings that show a significant positive role of temperature and to a lesser extent of disasters on short-distance internal migration. However, we do not find a clear evidence of a significant effect of rainfall on internal migration.

There are multiple channels through which climate may influence migration. We cannot study all possible channels, but given the significance of agriculture in the Philippines, we focus on examining the potential effect of climate on migration through its impact on agricultural productivity. We use rice yield as a measure of agricultural productivity for two reasons. First, rice is the most important crop in the Philippines in terms of land area occupied and value of production as of 2003.^11^ Second, rice is extremely sensitive to changing weather patterns. For example, rice growth and production are affected by extreme weather events such as more rainy days, warmer nights, and prolonged drought resulting in lower yield (Centeno and Wassmann 2010).

**Fig. 1:**
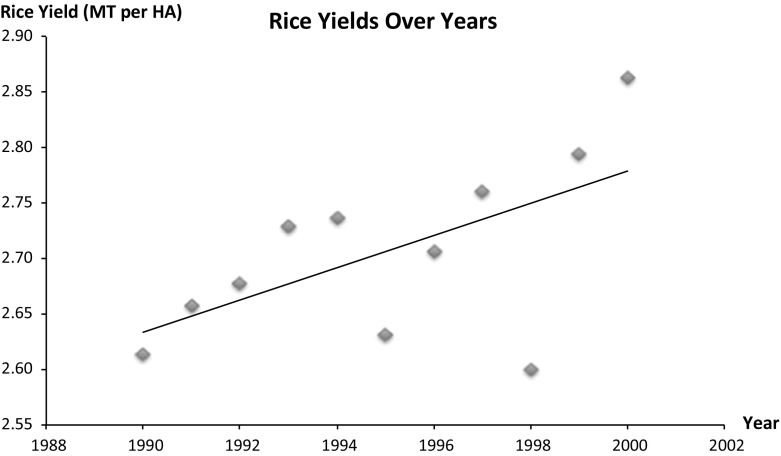
Rice yield over years

We predict the direct effects of the same climate variables used in our migration equation on rice yields using annual rice yield and climate data. We use rice yield (kg per Ha), converted to log of rice yield as a proxy for the overall agricultural yields. Since climate and yield have a direct year-to-year relationship, it is most appropriately studied at an annual time step, and therefore, we use yearly rice yield and climate data for this analysis. Nevertheless, the significance and direction of the effects of the climate variables on rice yield are consistent even when we use 5-year averages of the climate variables and the yield data for the analysis. Our review of the yearly rice yield data between 1990 and 2000 (See Fig. 1) suggests a linear time trend in rice yield with yields trending upward over time. In our yearly analysis, therefore, we control for a linear time trend in yield to control for technological improvement over time.

Table 3 summarizes results of our regression. Temperature has a significant negative effect on annual rice yield—each 1 °C increase in the summer temperature decreases annual total rice yield by approximately 6 %. Similarly, typhoon activity also consistently lowers annual total rice yield—each percentage increase in normalized death rates from typhoons decreases rice yield by 0.05 %. Once again, the standardized coefficients suggest a much stronger impact of temperature (compared to typhoons) on rice yield, which is consistent with our findings on climate–migration link.

**Table 3:** Climate effects on rice yield

Independent variables	Total annual rice yield	
	Actual coefficients	Standardized coefficients
Average summer temperature, April–September (°C)	−0.0611**	−0.2972**
	(0.026)	(0.125)
Average of maximum monthly precipitation in the wet season, July–December (m/yr)	−0.0133	−0.0076
	(0.041)	(0.023)
Average of minimum monthly precipitation in the dry season, January–June (m/yr)	0.2696	0.0458
	(0.227)	(0.038)
Deaths from typhoons (normalized by population of province)	−0.0448***	−0.0583***
	(0.017)	(0.022)
No. of observations	743	743
Adjusted *R*-squared	1.000	0.832

Regressions use annual rice yield and climate data. Also, logarithmic transformation of measure of rice yield and typhoons used. Results control for province fixed effects and a linear time trend in yield. Standard errors (in parentheses) corrected for spatial autocorrelation and within province clustering

*** *p* < 0.01; ** *p* < 0.05; * *p* < 0.1

As for the role of precipitation, consistent with its effect on migration, we did not find a significant effect of either maximum or minimum precipitation on the yield. Unlike the case of some other places like Burkina Faso, where rainfed agriculture is the main source of livelihoods in rural areas and where climate effects on migration have been studied (Henry et al. 2003, 2004), irrigated rice accounts for 75 % of the total rice produced in the Philippines. Furthermore, precipitation has more spatial heterogeneity, and as a result, it may be harder to capture its actual variability by the relatively coarse nature of the climate data (Lobell and Burke 2010; Burke et al. 2009; Cai et al. 2016). Consequently, it might be harder to capture the effect of rainfall on yields.

So far, we found that an increase in temperature as well as typhoon activity reduced rice yields, while temperature increase and to some extent typhoon activity also increased outmigration. On the other hand, the role of precipitation on migration is unclear and precipitation has no significant effect on rice yields either. Our results therefore hint that a decline in the yield in response to higher temperature and typhoon activity is likely to induce inter-provincial moves. If there is indeed an agricultural channel through which climate induces migration (through its impact on crop yields), then we would expect provinces that are more dependent on agriculture to be more sensitive to changes in climate (particularly an increase in temperature) in terms of their migration response, compared to provinces that are less dependent on agriculture.

Provinces with higher share of rural population are likely to be more dependent on agriculture as a source of livelihoods and income versus those with higher share of urban population. If it is indeed the agricultural channel through which temperature induces migration, then we would expect the former types of provinces to be more likely to send out migrants in response to changes in climate compared to the latter provinces.

Using data on the rural/urban composition of each province in 1990,^12^ we ranked provinces by their composition of rural population (measured by the percentage of people living in rural areas versus urban areas). We then divided provinces into “provinces with higher rural share” and “provinces with lower rural share.” We classified provinces with higher rural share as those that are above the 50th percentile compared to the rest of the provinces that are below the 50th percentile cutoff. This is consistent with approach used in previous study by Cai et al. (2016). Additionally, using a different cutoff of 25th percentile does not change our results by much. We then tested the migration sensitivity of more rural and less rural provinces to climate variables.

The results on the right-hand side of Table 2 reveal that among provinces with higher share of rural population, an increase in both temperature and typhoon activity significantly increases outmigration although the relative effect of temperature is much stronger than that of typhoon, which is consistent with our previous findings. On the contrary, among provinces with lower share of rural population, only typhoon activity seems to have a much smaller effect (and only marginally significant) on outmigration. These results, along with the earlier findings that established significant negative effects of temperature and typhoons on rice yield and positive effects on migration, suggest that the agricultural channel is one through which climate may influence migration, although not necessarily to the exclusion of other channels.

In order to further improve our understanding of the underlying mechanisms for the climate–migration link, we examined the climate–migration sensitivity for demographic and socioeconomic subgroups, testing whether the effects differ across subgroups. We used information on the characteristics of the population such as gender, age at the beginning of each five-year migration period, and level of education (in year 2000). In Table 4, we summarize the results.

**Table 4:** Climate effects on outmigration broken down by demographic characteristics

Independent variables	Gender				Age					
	Females		Males		20–29		30–49		50–60	
	Actual coefficients	Standardized coefficients	Actual coefficients	Standardized coefficients	Actual coefficients	Standardized coefficients	Actual coefficients	Standardized coefficients	Actual coefficients	Standardized coefficients
Average summer temperature, April–September (°C)	0.0052***	1.0388***	0.0068**	1.2059**	0.0105***	1.3448***	0.0040**	0.8859**	0.0041**	1.1785**
	(0.002)	(0.318)	(0.003)	(0.454)	(0.003)	(0.392)	(0.002)	(0.398)	(0.002)	(0.452)
Average of maximum monthly precipitation in the wet season, July–December (m/yr)	−0.0040	−0.0746	0.0098**	0.1656**	0.0050	0.0603	0.0003	0.0071	0.0121***	0.3293***
	(0.004)	(0.074)	(0.004)	(0.065)	(0.006)	(0.068)	(0.003)	(0.057)	(0.004)	(0.117)
Average of minimum monthly precipitation in the dry season, January–June (m/yr)	−0.0080	−0.0481	−0.0508*	−0.2772*	−0.0554*	−0.2169*	−0.0193	−0.1304	−0.0271	−0.2396
	(0.024)	(0.145)	(0.026)	(0.141)	(0.033)	(0.129)	(0.023)	(0.157)	(0.021)	(0.189)
Deaths from typhoons (normalized by population of province)	0.0010**	0.1448**	0.0016**	0.1994**	0.0021***	0.2066***	0.0007*	0.1111*	0.0006**	0.1752**
	(0.000)	(0.059)	(0.001)	(0.078)	(0.001)	(0.068)	(0.000)	(0.057)	(0.000)	(0.079)
No. of observations	138	138	138	138	138	138	138	138	138	138
Adjusted *R*-squared	0.981	0.813	0.970	0.732	0.974	0.752	0.973	0.0071	0.941	0.704

Independent variables	Education			
	Secondary and above		Primary and below	
	Actual coefficients	Standardized coefficients	Actual coefficients	Standardized coefficients
Average summer temperature, April–September (°C)	0.0085***	1.1150***	0.0045**	0.9126**
	(0.003)	(0.335)	(0.002)	(0.395)
Average of maximum monthly precipitation in the wet season, July–December (m/yr)	0.0077*	0.0953*	0.0050	0.0962
	(0.004)	(0.048)	(0.003)	(0.066)
Average of minimum monthly precipitation in the dry season, January–June (m/yr)	0.0075	0.0301	−0.0635***	−0.3961***
	(0.035)	(0.139)	(0.022)	(0.137)
Deaths from typhoons (normalized by population of province)	0.0022***	0.2351***	0.0011**	0.1503**
	(0.000)	(0.047)	(0.001)	(0.071)
No. of observations	138	138	138	138
Adjusted *R*-squared	0.982	0.814	0.961	0.756

Migration and climate/disaster variables are both measured as 5-year averages between the time periods of 1990 to 1995 and 1995 to 2000. Logarithmic transformation of measure of typhoons used. Results control for province and time (5-year) fixed effects. Standard errors (in parentheses) corrected for spatial autocorrelation and within province clustering

*** *p* < 0.01, ** *p* < 0.05, * *p* < 0.1

The results on climate–migration sensitivity for males versus females suggest that a rise in temperature significantly increases outmigration among both males and females, but the effects are higher among males, which is consistent with our hypothesis and along the lines with findings discussed earlier in Mueller et al. 2014. While each 1 °C increase in the summer temperature increases female outmigration rate by 0.5 % points, it increases male outmigration rate by 0.7 % points. Typhoons also have a higher significant positive impact on male migration (compared to female migration)—each percentage increase in the normalized death rates from typhoons increases male migration by 0.16 % points, while it increases female outmigration rate by 0.10 % points. Even negative rainfall outcomes (increase in maximum wet season precipitation and decrease in minimum dry season precipitation) result in significant increase in migration among males but have no significant impact on female migration, in lines with findings in Gray and Mueller (2012). The results suggest a potential economic channel such as a decline in agricultural productivity as a consequence of higher temperature, increased typhoon activity, and negative rainfall outcomes, which may induce men, the primary bread earners in patriarchal societies to migrate more than women.

Similarly, we explore migration sensitivity to climate of different age groups by breaking the sample into three categories: 20- to 29-, 30- to 49-, and 50- to 60-year-olds. Consistent with findings in Feng et al. (2013), we find that the youngest age group (20–29) is most likely to respond to an increase in temperature and incidents of typhoons by migrating. The migration sensitivity to climate seems to generally decline with age as evident from the coefficients of temperature and typhoons that become smaller for older age groups. While each 1 °C increase in the summer temperature increases outmigration rate of 20- to 29-year-olds by 1 % point, it increases the outmigration rate among 30- to 49-year-olds as well as among 50- to 60-year-olds by only 0.4 % points. Similarly, the effect of typhoons on migration clearly subsides with age with each percentage point increase in normalized death rates from typhoons increasing migration by 0.2 % points among the youngest age group, but by only 0.07 and 0.06 % points among the middle and oldest age groups, respectively. With precipitation, we find contradictory results, such that negative rainfall outcome (captured by a decrease in minimum dry season precipitation) results in significant increase in migration only among the youngest age group, which is in line with the effect of other climate variables. However, negative rainfall outcome (captured by an increase in maximum wet season precipitation) leads to significant increase in migration only among the oldest age group.

Finally, using an individual’s level of education as a proxy of their socioeconomic status as well as their level of financial and human capital, we test the sensitivity of population subgroups (based on their level of education) to climate variables. We predict the effects of climate variables on more educated (those with secondary education and more) versus less educated (those with primary education and less). Consistent with our hypothesis and findings discussed earlier, we find that the effects of climate variables on migration increase with education. While each 1 °C increase in the summer temperature increases outmigration among more educated by 0.85 % points, it increases outmigration of less educated by only 0.45 % points. Likewise, each percentage increase in the normalized death rates from typhoons increases migration of more educated by 0.22 % points, but it increases migration of less educated by only 0.11 % points. Even maximum wet season precipitation has a positive significant effect on increasing migration among the more educated but has no significant effect on the migration outcome of less educated.

These results are thus generally consistent with our hypothesis, with the exception that negative precipitation outcome (captured by a decline in minimum monthly dry season precipitation) seems to significantly increase migration of less educated but has no significant effect on more educated. The results, in general, are also consistent with some new studies that challenge the generality of the positive link between environment and migration, suggesting that in the case of poor and vulnerable or those from lower socioeconomic status, negative climate shock may actually constrain movement.

## Conclusion

Using a panel data structure, we explore the effects of climatic variations and extremes on inter-provincial migration within the Philippines. We find that temperature and to some extent increased typhoon activity induce outmigration with the effects being driven mostly by migration of males, more educated, and younger individuals. The climate effect on gender is somewhat contradictory to the general trend of higher outmigration among females than males in the Philippines and underscores the importance of other social and economic drivers of migration, which might dominate the climate effect. The extremes of precipitation on the other hand do not have a consistent or significant effect on migration across the different regressions. In order to better understand the underlying mechanism through which climate may influence migration, we use rice yield as a proxy measure of agricultural productivity. Our findings show that consistent with our results of a significant positive effect of temperature and typhoons on migration, the same climate variables also have a significant negative effect on rice yield. We further find evidence of a significantly higher outmigration from provinces with higher share of rural population compared to those with lower share of rural population.

Our paper contributes to the limited literature that investigates the effects of natural disasters and climatic variations on migration and further adds to the limited findings on climate–migration sensitivity across demographic subgroups. The findings  on agriculture acting as an intermediate link, and higher migration among males, educated, and younger people are consistent with the migration theory that a negative shock to income through the agricultural sector can promote migration of those with higher earning potential: the more educated (given their higher level of human capital); younger people (with more years to enjoy the increased earnings); and males (primary bread earners, with less household responsibilities). Lastly, the finding has implications for future effects of global warming on migration, pointing toward the possibility of more outmigration from places that already have a high baseline temperature.

## Footnotes

1 Given the nature of the data we exploited, we limit our analysis in this study to internal migration.

2
http://countrystat.bas.gov.ph/?cont=3.

3
http://www.worldwatch.org/node/558.

4 Primary administrative seats of rural districts in the Philippines.

5 Refers to studies analyzing individual migration.

6 Refers to studies analyzing aggregate migration.

7
https://international.ipums.org.

8
http://countrystat.bas.gov.ph/.

9
http://dbmp.philrice.gov.ph/Ricestat/Statmonth%20data/Volume_1prod.html.

10
http://www.desinventar.net/DesInventar/main.jsp.

11
http://www.fao.org/ag/agp/AGPC/doc/Counprof/Philippines/Philipp.htm.

12 Data from IPUMS available only for 1990 and not for 1995.
